# Alternative Treatment for Asthma: Case Study of Success of Traditional Chinese Medicine Treatment of Children from Urban Areas with Different Levels of Environmental Pollution

**DOI:** 10.5402/2012/547534

**Published:** 2012-08-09

**Authors:** Helen Kopnina

**Affiliations:** The Hague University of Applied Science, 2521 EN The Hague, The Netherlands

## Abstract

The present study examined efficacy of traditional Chinese medicine (TCM) treatment in Dutch children with asthma in areas with differing air pollution. The study results indicate that TCM treatment of children living in more polluted urban area is less successful then that of children living in cleaner air area.

## 1. Introduction

### 1.1. Use of Traditional Chinese Medicine (TCM) in Patients with Asthma

In the Chinese, Japanese, Korean, Indian, and Western cultures, herbal therapies appear to be commonly used for asthma. Well-controlled scientific studies have not been performed on many of the Asian herbal therapies, and some basic studies have been performed on various herbal components (active ingredients); more needs to be done to assess the composite effects of many herbal remedies [1]. Complementary and alternative medicine (CAM) therapies such as traditional Chinese medicine (TCM), ayuverdic medicine, herbal therapy, acupuncture, yoga, homeopathy, chiropractic medicine, and massage therapy continue to gain popularity as modalities for the treatment of asthma in Western Europe in general and in The Netherlands in particular. CAM is commonly defined as a group of diverse medical and healthcare systems, practices, and products that are not generally considered part of conventional medicine. Complementary medicine is used together with conventional medicine, and alternative medicine is used in place of conventional medicine (National Center for Complementary and Alternative Medicine NCCAM). 

CAM use is widespread because parents are seeking a cure for asthma, as well as alternative methods that are natural, without long-term side effects [2, 3]. In The Netherlands, CAM in general and TCM in particular are still not officially recognized as a viable alternative by most medical practitioners, pharmaceutical and insurance companies. The medical practitioners, themselves educated within the dominant paradigm, continue to offer the patients pharmaceutical-based medicine as the most effective and scientifically validated form of medicine [4]. These medicines typically contain corticosteroids and beta-agonists, the long-term use of which indicates serious side effects. State-supported pharmaceutical industry and insurance companies largely subsidize delivery of these medicines, while CAM delivery is largely funded privately. It is perhaps not surprising that asthma patients' noncompliance to the prescribed medical regime is seen by most practitioners as a matter of “denial”-either of rationality guiding the evidence-based medicine or of the patients' own identity as asthma sufferers. 

Li and Brown [5] analyzed the literature regarding the care of asthmatic children using biologically based CAM therapies and identified three clinical studies involving children and antiasthma TCM herbal remedies. The three specific TCM formulas were the following: modified Mai Men Dong Tang (mMMDT) consisted of five herbs, Ding Chuan Tang (DCT) comprised of nine herbs, STA-1 consisted of a combination of mMMDT (10 herbs). In all three studies, the subjects continued their prescribed medical regimen and were either given a TCM formula or a placebo. The treatment subjects demonstrated improved forced expiratory volume (FEV1) outcome, compared to the subjects given a placebo in all three studies. All participants were able to tolerate the TCM formulas safely.

Another study utilizing the principles of TCM applied Sanfujiu to treat allergies and asthma by increasing yang qi (nature of the sun; hot) in the lungs. Sanfujiu is a paste that consist of five Chinese medicines Tai et al. [6]. This study enlisted 119 subjects of all ages. Those with asthma were more likely to report that the Sanfujiu treatment was effective compared to subjects with other allergic diseases.

Stockert et al.'s [7] study examined the effectiveness of a combination of laser acupuncture and probiotics as a form of treatment for asthma was reviewed. A small group of 17 children was enlisted to participate in a randomized, placebo-controlled, double-blind study that treated asthmatic children with TCM methods. Laser acupuncture was substituted for needle acupuncture, and probiotics of nonpathogenic *Enterococcus faecalis *was administered in place of the TCM herb, Jin Zhi. The control group was treated with a laser pen and given placebo drops to ingest. The results of the study demonstrated that the treatment group had significantly decreased weekly peak flow variability as a measure of bronchial hyperreactivity [7]. As the recent study of Liu and Gong [8] shows, acupuncture can also have a remarkable effect in stopping an acute asthma attack.

Arnold et al. [9] addressed efficacy and safety of herb- and plant-based preparations for treatment of asthma. Primary outcomes led authors to conclude that evidence base for the effects of herbal treatments is hampered by the variety of treatments assessed, poor reporting quality of the studies, and lack of available data. Positive findings in this review warrant additional well-designed trials in this area [9].

It is remarkable that no consistent clinical trials aside from rather patchy ad hoc studies have been conducted up till present. An important part of the assessment of CAM modalities is the therapeutic-toxicologic safety profile (risk-benefit ratio), and further research evaluating the clinical efficacy and mechanism of action of various CAM interventions for asthma is greatly needed [10]. Li and Brown [5] and Mainardi et al. [11] summarized the difficulties of testing biologically based TCM modalities as the following:

1. isolation and identification of active constituents may be difficult because of nature of the herbs and its manufacture and preparation processes;
2. synergistic effect of herbal combinations complicates the ability to determine the exact effect;
3. TCM formulas are traditionally created for the individual; standardization of TCM may not be effective; random, blinded studies are difficult to conduct when the subject's perception of CAM therapies may influence the results;
4. there is a need for more controlled clinical trials to test the efficacy of TCM formulas [5, 11].

Additional hypothesis for the lack of conclusive evidence on efficiency and safety of CAM may be due to the fact that the type of funding may have determinant effects on the design of studies and on the interpretation of findings [4]. Funding by the industry is associated with design features less likely to lead to finding statistically significant adverse effects and with a more favorable clinical interpretation of such findings. Disclosure of conflicts of interest should be strengthened for a more balanced opinion on the safety of drugs [12]. 

At present, there are few consistent Western-based studies that address clinical trials of CAM (while there are quite a few published in other languages, such as Chinese). Social scientists and mainstream Western practitioners alike continue to disregard CAM as a serious alternative to potentially harmful conventional medicine [13]. Astma Fonds, official Asthma patients organization in The Netherlands, refuses to place any materials related to CAM on its site.

### 1.2. Asthma and Air Pollution

Recent studies show a relationship between exposure to air pollutants for both the occurrence of the disease and exacerbation of childhood asthma [14]. There is growing evidence of asthma symptoms in children who live near roadways with high traffic counts [15–21]. These recent international studies provide the evidence for traffic pollution as a risk factor for both asthma exacerbation and onset as strong. The recent population-based matched case-control study of Li et al. [22] examined the relationship between air pollution and severity of asthma symptoms. Asthma events were associated with traffic pollution and proximity to primary roads, providing moderately strong evidence of elevated risk of asthma close to major roads. Particularly traffic pollutants were linked to higher occurrences of asthma [23–25]. Another study of urban air pollution and emergency room admissions for respiratory symptoms in Italy demonstrates that exposure to ambient levels of air pollution is an important determinant of emergency room (ER) visits for acute respiratory symptoms, particularly during the warm season. ER admittance may be considered a good proxy to evaluate the adverse effects of air pollution on respiratory health [26].

### 1.3. Air Pollution in The Netherlands

**Figure 1:**
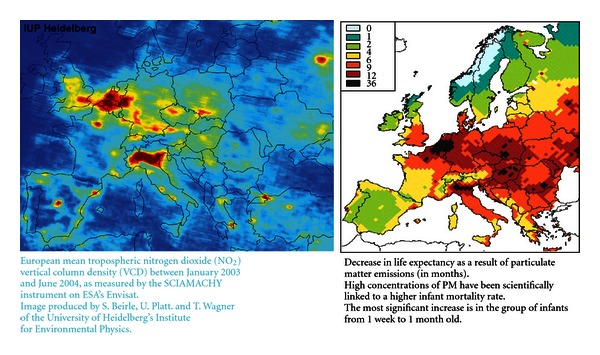


The Netherlands relies for 92% on fossil sources of primary energy. Emissions and waste include carbon monoxide (CO); particulate matter (PM10, PM2,5); nitrates (NO*x*); sulfates (SO*x*); heavy metals (As, Cd, Cr-VI, Ni, Hg, Pb); volatile organic components (VOCs); polycyclic aromatic Carbohydrates (PACs). For major pollutants and their effect on health, see Table 4 in the appendix section.  Figure 1 shows pollution density in Europe. It appears that The Netherlands is one of the most polluted countries in Western Europe.

With more than seven million passenger vehicles on its roads, The Netherlands is the sixth largest automotive market in Europe [27]. According to Eurostat [28], car density in The Netherlands is 460 per 1000 inhabitants, up from 371 per 1000 in 1991. This is remarkable, because The Netherlands is a small country with a highly developed public transportation system. The Dutch railway network had a traffic performance of more than 20 thousand kilometres of railroad—twice the EU average—per kilometre of track in 2006.

According to the national research on mobility in The Netherlands (Mobiliteitsonderzoek Nederland (MON) published in 2010, there are 7,348 million households. Four out of five (79,1%) own one or more cars, 20,9% does not own a car. There are very few carless families (4%), and four in five single parent families own a car. According to the recently published dissertation of Hans Jeekel, titled “The Car-Dependent Society” (De autoafhankelijke samenleving), 20% of the carless persons can simply not afford a car. In an interview to the Dutch newspaper Jeekel [29], Jeekel pronounced: “Car ownership is becoming a pre-requisite for social acceptance. Employees expect employees to travel by car, increasing number of residential and commercial areas are positioned along the highway. Working mothers ride to and fro between office, school, sport club, and the supermarket, all within a few hours”. Together with other air pollutants, traffic pollution contributes significantly to the high occurrence of asthma in The Netherlands.

### 1.4. Asthma in The Netherlands

According to the Dutch Ministry of Public Health RIVM, there are 519.800 (236.800 men and 283.000 women) “official” asthma patients in The Netherlands, among whom 115.000 are children (last statistics available from 2003). In the Dutch cross-sectional study examining whether motor vehicle exhaust from freeways has an effect on respiratory health of children, 1068 children attending schools situated less than 1000 m from major freeways carrying between 80,000 and 150,000 vehicles per day in the Province of South Holland were asked to participate. Chronic respiratory symptoms reported in the questionnaire were analyzed with logistic regression. Distance from the freeway and (truck) traffic intensity was used as exposure variables. Cough, wheeze, runny nose, and doctor-diagnosed asthma were significantly more often reported for children living within 100 m from the freeway. Truck traffic intensity and the concentration of black smoke measured in schools were found to be significantly associated with chronic respiratory symptoms [15]. Remarkably, environmental awareness of the adverse effects of personal vehicle pollution, environment, and health in Dutch society is very low [30].

In the recent report published by the University of Utrecht and Astma Fonds, 45% of 8-year-old patients has “insufficient” medical regime. According to the report of the Astma Fonds, parents are either insufficiently informed about the use of medication (in case of parents with low education) or seem to refuse to use the medication for “fear of side effects.” (Online sources available to the patients and supplied to the author of this article by the patients themselves included warnings on the medicine labels indicating risk of asthma-related death. For example, for Symbicort, the following is known. A 28-week, placebo-controlled US study comparing the safety of salmeterol with placebo, each added to usual asthma therapy, showed an increase in asthma-related deaths in patients receiving salmeterol (13/13,176 in patients treated with salmeterol versus 3/13,179 in patients treated with placebo; RR 4.37, 95% CI 1.25, 15.34). The increased risk of asthma-related death may represent a class effect of the long-acting beta_2_-adrenergic agonists, including formoterol. No study adequate to determine whether the rate of asthma-related death is increased with SYMBICORT has been conducted (enclosed with the medicine, “Warnings”). A recent meta-analysis of the roles of long-acting beta-agonists may indicate a danger to asthma patients: long-acting  beta_2_-adrenergic agonists may increase the risk of asthma-related death… Data from a large placebo-controlled US study that compared the safety of another long-acting  beta_2_-adrenergic agonist (salmeterol) or placebo added to usual asthma therapy showed an increase in asthma-related deaths in patients receiving salmeterol. This finding with salmeterol may apply to formoterol (a long-acting  beta_2_-adrenergic agonist), one of the active ingredients in SYMBICORT… Clinically significant cardiovascular effects and fatalities have been reported in association with excessive use of inhaled sympathomimetic drugs. (http://www.rxlist.com/symbicort-drug.htm). While the fear of side effects is seen as irrational by most main-stream Western asthma specialists, earlier research among Dutch asthma patients has demonstrated that it is particularly highly educated and well-informed patients who choose CAM medicine as an alternative to conventional inhalators containing corticosteroids and beta-agonists [1, 31]. 

## 2. Case Study

However, the efficacy of CAM and TCM is largely disputed, especially in the case of asthma caused by severe air pollution factors. The case study conducted involved a sample of 47 Dutch children between 7 and 13 years old who were diagnosed with asthma. There were two groups of children whose parents refused to use conventional medicine and resorted to TCM (often in combination with other types of CAM therapy such as acupuncture) and one group that used conventional medicine.

First group (case study 1; see Table 1) of children—*n* = 16, lived in proximity of major highway, second group (case study 2; see Table 2)—*n* = 20 lived in a suburban “green” area with little traffic. It needs to be noted that the first group came from mostly low-income families of ethnically heterogeneous origin (there were 6 Moroccan, 2 Antillean, 2 Turkish, and 6 Dutch children). Another group of children came from mostly Dutch well-to-do family backgrounds. A group of 11 children (case study 3; see Table 3) living in the same urban area (close to highway) as group 1 but using conventional medication was used as control group.

The children were followed for one year (between January 2010 and January 2011), and their parents were asked to complete self-report “health diary” forms, the results of which are summed up in in Tables 1, 2, and 3. 

## 3. Reflection

The sample is too small to make any definite conclusions; however, if any generalization is to be made, it appears that the children from the more air-polluted area showed fewer positive effects of TCM. A salient detail is that, among twenty well-to-do families of the second group, almost all owned at least one family car (18 families) as opposed to only 6 car-owners from the first group. During the research on perception of environmental and health effects of car ownership, it appeared however that lower-income families expressed a great interest in owning a car as soon as they were financially able to [30]. While the parents of the second group expressed greater awareness of facts about asthma and asthma medication, practically no family members from either the first or the second and third group expressed awareness of adverse effects of traffic pollution on asthma. 

## 4. Conclusion

It may be too premature to draw conclusions based on this small-sample pilot study, yet recommendation can be made that TCM may not be as effective in treating childhood asthma if it is caused by or exacerbated by residence in air-polluted areas. Conventional medicine seemed to have consistent effect on symptom reduction in children with polluted areas. However, concerns about safety of long-term use of conventional medicines remain. The study also raises questions about efficacy of asthma treatment if measures addressing general air pollution and traffic in particular are not taken.

## Figures and Tables

**Table 1:** Case study 1. Children from the highway area using TCM (*n* = 16).

Self-report	1–3 month	4–6 months	7–9 months	9–12 months
Codes (1) No improvement	6 families: 1	7 families: 1	6 families: 1	7 families: 1
(2) Some improvement	6 families: 2	5 families: 2	5 families: 2	5 families: 2
(3) Good improvement	4 families: 3	2 families: 3	1 family: 6	4 families stopped with therapy of refused or report
(4) Stopped with therapy due to success		1 family: 5	3 families: 5	
(5) Stopped with therapy due to failure (taken up conventional therapy)		1 family: refused to report	1 family: refused to report	
(6) Stopped with therapy due to failure (using nothing or data missing)				

**Table 2:** Case study 2. Children from the park area using TCM (*n* = 20).

Self-report	1–3 month	4–6 months	7–9 months	9–12 months
Codes (1) No improvement	6 families: 1	5 families: 1	3 families: 1	3 families: 1
(2) Some improvement	10 families: 2	11 families: 2	11 families: 2	9 families: 2
(3) Good improvement	4 families: 3	4 families: 3	4 families: 3	6 families: 3
(4) Stopped with therapy due to success			1 family: 4	1 family: 4
(5) Stopped with therapy due to failure (taken up conventional therapy)			1 family: refused to report	1 family: refused to report
(6) Stopped with therapy due to failure (using nothing or data missing)				

**Table 3:** Case study 3. Children from the highway area using conventional medicine (*n* = 11).

Self-report	1–3 month	4–6 months	7–9 months	9–12 months
Codes (1) No improvement	2 families: 1	1 family: 1	1 family: 1	5 families: 2
(2) Some improvement	5 families: 2	5 families: 2	5 families: 2	6 families: 3
(3) Good improvement	4 families: 3	4 families: 3	4 families: 3	
(4) Stopped with therapy due to success				
(5) Stopped with therapy due to failure (taken up conventional therapy)				
(6) Stopped with therapy due to failure (using nothing or data missing)				

**Table 4:** Air pollutants and their effects on health.

Primary pollutants	Secondary pollutants	Impacts
Particles (PM_10_, PM_2.5_, black smoke)		Mortality Cardio-pulmonary morbidity (cerebrovascular hospital admissions, congestive heart failure, chronic bronchitis, chronic cough in children, lower respiratory symptoms, cough inasthmatics)
SO_2_		Mortality
		Cardio pulmonary morbidity (hospitalisation, consultation of doctor, asthma, sick leave, restricted activity)
SO_2_	Sulphates	Like particles?
NO*x *		Morbidity?
NO*x *	Nitrates	Like particles?
NO*x* + VOC	Ozone	Mortality Morbidity (respiratory hospital admissions, restricted activity days, asthma attacks, symptom days)
CO		Mortality (congestive heart failure) morbidity (cardiovascular)
PAH		Cancers
diesel soot, benzene,	
1,3-butadiene, dioxins	
As, Cd, Cr-VI, Ni		Cancers Other morbidity
Hg, Pb		Morbidity (neurotoxic)

## Appendix

For more details, see Table 4.
